# Synthesis of Mn_0.5_Zn_0.5_Sm_x_Eu_x_Fe_1.8−2x_O_4_ Nanoparticles via the Hydrothermal Approach Induced Anti-Cancer and Anti-Bacterial Activities

**DOI:** 10.3390/nano9111635

**Published:** 2019-11-18

**Authors:** Sultan Akhtar, Suriya Rehman, Munirah A. Almessiere, Firdos Alam Khan, Yassine Slimani, Abdulhadi Baykal

**Affiliations:** 1 Department of Biophysics, Institute for Research and Medical Consultations (IRMC), Imam Abdulrahman Bin Faisal University, P.0. Box 1982, Dammam 31441, Saudi Arabia; suakhtar@iau.edu.sa (S.A.); malmessiere@iau.edu.sa (M.A.A.); yaslimani@iau.edu.sa (Y.S.); 2 Department of Epidemic Disease Research, Institute for Research and Medical Consultations (IRMC), Imam Abdulrahman Bin Faisal University, P.0. Box 1982, Dammam 31441, Saudi Arabia; surrehman@iau.edu.sa; 3 Department of Physics, College of Science, Imam Abdulrahman Bin Faisal University, P.0. Box 1982, Dammam 31441, Saudi Arabia; 4 Department of Stem Cell Biology, Institute for Research and Medical Consultations (IRMC), Imam Abdulrahman Bin Faisal University, Post Box No. 1982, Dammam 31441, Saudi Arabia; fakhan@iau.edu.sa; 5 Department of Nano-Medicine Research, Institute for Research and Medical Consultations (IRMC), Imam Abdulrahman Bin Faisal University, P.0. Box 1982, Dammam 31441, Saudi Arabia

**Keywords:** spinel ferrites, cytotoxicity, antibacterial activity, hydrothermal synthesis

## Abstract

Manganese metallic nanoparticles are attractive materials for various biological and medical applications. In the present study, we synthesized unique Mn_0.5_Zn_0.5_Sm_x_Eu_x_Fe_1.8−2x_O_4_ (0.01 ≤ x ≤ 0.05) nanoparticles (NPs) by using the hydrothermal approach. The structure and surface morphology of the products were determined by X-ray powder diffraction (XRD), transmission electron and scanning electron microcopies (TEM and SEM), along with energy dispersive X-ray spectroscopy (EDX). We evaluated the impact of Mn_0.5_Zn_0.5_Sm_x_Eu_x_Fe_1.8−2x_O_4_ NPs on both human embryonic stem cells (HEK-293) (normal cells) and human colon carcinoma cells (HCT-116) (cancerous cells). We found that post-48 h of treatment of all products showed a significant decline in the cancer cell population as revealed by microscopically and the (3-(4,5-dimethylthiazol-2-yl)-2,5-diphenyltetrazolium bromide) tetrazolium (MTT) assay. The inhibitory concentration (IC_50_) values of the products ranged between 0.75 and 2.25 µg/mL. When tested on normal and healthy cells (HEK-293), we found that the treatment of products did not produce any effects on the normal cells, which suggests that all products selectively targeted the cancerous cells. The anti-bacterial properties of the samples were also evaluated by Minimum Inhibitory Concentration (MIC) and Minimum Bactericidal Concentration (MBC) assays, which showed that products also inhibited the bacterial growth.

## 1. Introduction

In recent years, spinel ferrites have attracted much attention from researchers due to their versatile magnetic properties and wide range of applications. Nanosized ferrites have shown enhanced optical and magnetic properties compared to their bulk counterpart because of better magnetic coupling between the sub-lattices (tetrahedral (Td) or octahedral (Oh)) [1]. Due to improved properties, the nanosized ferrites have been used for a broad range of applications, including: Fundamental research into industrial (sensors, refrigeration, transformers, electrical devices, disk recording, etc.) and medical applications (drug delivery, MRI (magnetic resonance imaging) technology separation, cancer therapy, biosensing, etc.) [2,3,4,5,6,7,8,9,10]. Ferrites are also most frequently used as magnetic absorbing materials owing to their cost effectiveness, excellent magnetic loss, and good stability [8]. Another advantage of nanosized spinel ferrites is that one can tune the magnetic properties by optimizing the size of the nanoparticles. A general structure of spinel ferrites is MFe_2_O_4_ (M = manganese, cobalt, zinc, magnesium, etc., Co, Zn, Mg, etc.). In this crystalline structure, anions (oxygen) form face-centered cubic arrangements and metal cations (M^2+^ and Fe^3+^) occupy either Td or Oh interstitial sites [11]. From the available ferrites, manganese-based ferrites (MnFe_2_O_4_) are considered as more significant because of their high Ms (saturation magnetization) and low loss of power at high frequencies [12]. Manganese ferrites are widely studied owing to their wide range of applications, such as in microwave industries, protecting the barrier against the harm of microwave, satellite communication, microwave dark room, etc. [13]. Furthermore, nanosized ferrites can provide better communication at high frequencies (10 kHz to a few MHz) with great stability because of their high electrical resistivity [13,14]. Recently, Co-Zn-substituted spinel-type ferrites have been studied and found attractive for bio-medical applications because of their suitable room temperature magnetic properties [15,16]. Spinel ferrites have also multiferroic applications. Multiferroic nanostructures have attracted lots of attention by researchers due to their unique benefits compared with bulk multiferroics. The interface between the piezoelectric phase and the ferromagnetic phase is well established and leads to a high magnetoelectric coupling effect by reducing the coupling loss [17,18].

Nanoparticles (NPs) possessed tremendous biological applications [19,20,21,22,23] as they have a dimension of less than 100 nm. There are various types of NPs, like metallic NPs, ceramic NPs, and polymeric NPs, and this classification is based on their size, shape, and property [19,23,24]. Over the past few years, metallic nanoparticles (MNPs) have been used in the field of medicine and pharmaceutical sciences [22,24]. There are many approaches, which have been developed to synthesize diverse and customized MNPs that can be used in experimental medicine, drug design, drug delivery, electrical and electronic engineering, electrochemical sensors, and biochemical sensors [23,24]. There has been an enormous increase in the rate of cancer occurrence around the world, and due to the non-availability of robust treatment, the rate of cancer morality has also been increasing. In this background, there is the need for an alternative approach to tackle this issue. MNPs possess better capabilities to treat the cancer as MNPs have an enhanced reactive surface area, which improves the therapeutic efficacy of the treatment.

To the best of our knowledge, Mn_0.5_Zn_0.5_Eu_x_Sm_x_Fe_1.5−2x_O_4_ (0.01 ≤ x ≤ 0.05) NPs have been synthesized (hydrothermally) and characterized, and its cytotoxicity and antibacterial activity was studied for the first time in this study. In this paper, we synthesized unique Mn_0.5_Zn_0.5_Eu_x_Sm_x_Fe_1.5−2x_O_4_ (0.01 ≤ x ≤ 0.05) NPs using the hydrothermal procedure. Both the spinel structure and nanosized particles were determined via XRD. The cubic morphology of them was proven by SEM, TEM, EDX, and elemental mapping spectroscopy. We evaluated the impact of as-synthesized NPs on two cancer cell lines: HCT-116 (human colorectal carcinoma cells) and HEK-293 (human embryonic kidney cells). The anti-bacterial properties of Mn_0.5_Zn_0.5_Eu_x_Sm_x_Fe_1.5−2x_O_4_ (0.01 ≤ x ≤ 0.05) NPs were also evaluated on *Escherichia coli* (*E. coli*) and *Staphylococcus aureus* (*S. aureus)* cells using Minimum Inhibitory Concentration (MIC) and Minimum Bactericidal Concentration (MBC), assays and SEM examination.

## 2. Experimental Procedure

### 2.1. Synthesis of Spinel Nanoparticles

Manganese (II) nitrate (Mn(NO_3_)_2_), zinc (II) nitrate (Zn(NO_3_)_2_), iron (III) nitrate hexahydrate (Fe(NO_3_)_3_.6H_2_O), europium(III) nitrate (Eu(NO_3_)_3_), and samarium(III) nitrate hexahydrate (Sm(NO_3_)_3_·6H_2_O) were utilized to fabricate Mn_0.5_Zn_0.5_Eu_x_Sm_x_Fe_1.5−2x_O_4_ (0.01 ≤ x ≤ 0.05) NPs via the hydrothermal approach. Specific ratios of metals nitrates were thawed in 50 mL of DI H_2_O with forceful stirring for 45 min. The pH was attuned at 11 by the addition of sodium hydroxide (NaOH) with stirring for 30 min and then the solution was exposed to an ultrasonic water bath for 40 min. The mixture was transferred to a Teflon-lined vessel, which was heated at 180 °C for 10 h in an oven. The final powder was washed with DI H_2_O several times and left to dry at 80 °C for 5 h. Structure was confirmed using XRD (Rigaku Benchtop Miniflex XRD analyzer with Cu Kα radiation) over the 2θ range of 20° to 70°. The microstructure was imaged by scanning electron microscope (SEM) and (TEM) (FEI Titan ST) coupled with energy-dispersive X-ray spectroscopy (EDX).

### 2.2. Anticancer Activities

#### 2.2.1. In Vitro Testing of Cytotoxicity

In the present study, cancer cell line (human colon carcinoma cells-HCT-116) and normal healthy cell line human embryonic kidney cells (HEK-293) were used to evaluate the cytotoxicity. The cells were cultured as per the method described previously [22,24]. In brief, cells were grown in DMEM media, L-glutamine, fetal bovine serum, selenium chloride, and antibiotic penicillin and streptomycin respectively in a CO_2_ incubator at 37 °C. When cells become 70% to 80% confluence, they were tested for the MTT (3-[4,5-dimethylthiazol-2-yl]-2,5-diphenyl-tetrazolium bromide (Molecules, New Zealand) assay, which was used to examine the impact of nanoparticles on cancer cell viability. Cells were treated with different concentrations (2.0 to 40 µg/mL) of Mn_0.5_Zn_0.5_Sm_x_Eu_x_Fe_1.8−2x_O_4_ (0.01 ≤ x ≤ 0.05) NPs. In the control group, we did not add Mn_0.5_Zn_0.5_Sm_x_Eu_x_Fe_1.8−2x_O_4_ (0.01 ≤ x ≤ 0.05) NPs. After 48 h of treatment, the culture medium was removed and 5.0-µL MTT (Sigma-Aldrich, St. Louis, MO, USA) solution (10 mg/mL) was added to each well and culture plates were incubated for 4 h. Then, culture media was removed and dimethyl sulfoxide (DMSO) was added in each well where MTT developed formazan crystals. Subsequently, culture plates were read under a microplate reader (Bio-Rad Laboratories, Hercules, CA, USA) at 570 nm. The data were analyzed with GraphPad Prism, GraphPad Software by a one-way analysis of variance (ANOVA) and *p*-values were calculated by Student′s t-test.

#### 2.2.2. Nuclear Staining by DAPI

The cancerous cells were stained with DAPI to examine the impact of Mn_0.5_Zn_0.5_Sm_x_Eu_x_Fe_1.8−2x_O_4_ (0.01 ≤ x ≤ 0.05) NPs on the cell nucleus. In the first group, HCT-116 cells were divided into two types: Group one was the control (without Mn_0.5_Zn_0.5_SmEuFe_1.8−2x_O_4_ (0.01 ≤ x ≤ 0.05) NPs treatment, and group two was the Mn_0.5_Zn_0.5_SmEuFe_1.8−2x_O_4_ (0.01 ≤ x ≤ 0.05) NPs-treated groups. Similarly, we also tested HEK-293 cells. They were divided into two groups: One was the control (without NPs treatment), and another one was NPs-treated groups. After 48 h of treatment, both cancerous and normal cells were pre-treated with ice-cold (4%) paraformaldehyde. Then, cells were treated with (0.1%) Triton X-100 in phosphate-buffered saline (PBS) for 5 min for cell membrane permeabilization. Both control and NPs-treated cells were stained with DAPI (1 μg/mL) prepared in PBS for 5 min in a dark environment. Finally, the cells were washed with (0.1%) Triton X-100 prepared in PBS. The nuclear morphology of both control and NPs-treated cells was examined under a confocal scanning microscope (Zeiss, Germany) equipped with a digital camera.

### 2.3. Antibacterial Activity

#### 2.3.1. Preparation of Test Nanomaterial and Inoculum

The NPs were homogenized and dissolved in sterile LB (Luria Bertaini) at a concentration of 16 to 0.5 mg/mL. For the preparation of the inoculum, test strains, i.e., gram negative (*Escherchia coli* ATCC35218) and gram positive (*Staphylococcus aureus* ATCC29213) were grown in LB overnight at 37  °C. The turbidity of the culture broth was adjusted to 10^6^ CFUs/mL using phosphate saline buffer (PBS).

#### 2.3.2. Minimal Inhibitory Concentration (MIC)

The MIC of the products was tested in the concentration ranging from 16 to 0.5 mg/mL, using the broth dilution method. The freshly adjusted bacterial inoculum was added to the prepared NP solution at a cell density of 2.5 × 10^5^ CFU mL^−1^ and further incubated at 35 ± 2 °C for 24 h with aeration. Untreated bacteria were included in the experiment as the negative control. The MIC was recorded as the lowest concentration of a drug, which visually inhibits 99% of bacterial growth [25].

#### 2.3.3. Minimal Bactericidal Concentration (MBC)

In continuation to the MIC evaluation of the NPs, an aliquot of incubated suspension with no apparent bacterial growth was plated on freshly prepared Mueller Hinton Agar (MHA) plates and further subjected to incubation at 35 ± 2 °C for 24 h. The MBC was recorded as the minimum concentration of a drug that killed 100% or having less than three CFU bacterial cells on the MHA plates [25].

#### 2.3.4. Effects of Mn_0.5_Zn_0.5_Sm_x_Eu_x_Fe_1.8−2x_O_4_ (0.01 ≤ x ≤ 0.05) NPs on the Morphology of Bacteria

The effects of Mn_0.5_Zn_0.5_Sm_x_Eu_x_Fe_1.8−2x_O_4_ (0.01 ≤ x ≤ 0.05) NPs on the morphology of *E. coli* and *S. aureus* cells were performed by SEM analysis as previously reported by Rehman et al. 2019 [25]. Precisely, ∼10^6^ CFU/mL of freshly grown bacterial cells treated with NPs (at the concentration obtained as MIC) were incubated with agitation at 37 °C overnight. The untreated bacteria were included in the experiment as a negative control. After the incubation period, cells were harvested by centrifugation at 12,000 rpm for 10 min. The cell pellets were washed using PBS and subsequently fixed with 2.5% glutaraldehyde for primary fixation, which was followed by secondary fixation, i.e., 1% osmium tetroxide. The fixed cells were washed and dehydrated by varying concentrations of a series of ethanol. Later, the cells were placed on the aluminum stubs, followed by drying in a desecrator, and finally coated with gold. Samples were photocaptured and analyzed at an accelerating voltage of 20 kV by SEM.

## 3. Results and Discussion

### 3.1. Structural Analysis and Morphological Study

**Figure 1:**
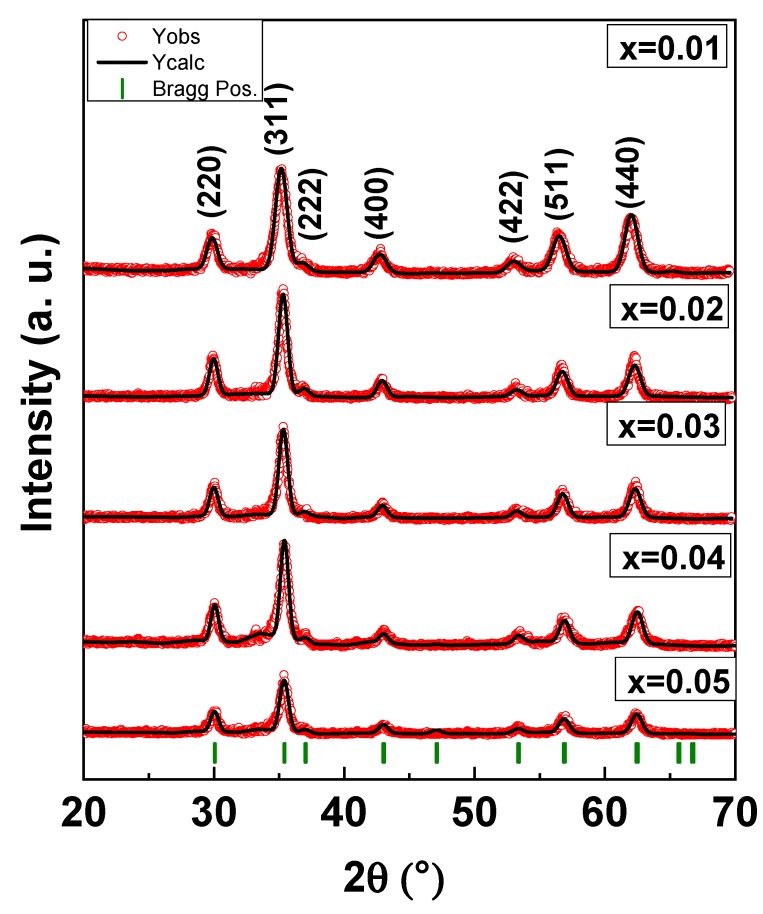
XRD powder patterns of Mn_0.5_Zn_0.5_Eu_x_Sm_x_Fe_1.5−2x_O_4_ (0.01 ≤ x ≤ 0.05) NPs.

**Figure 2:**
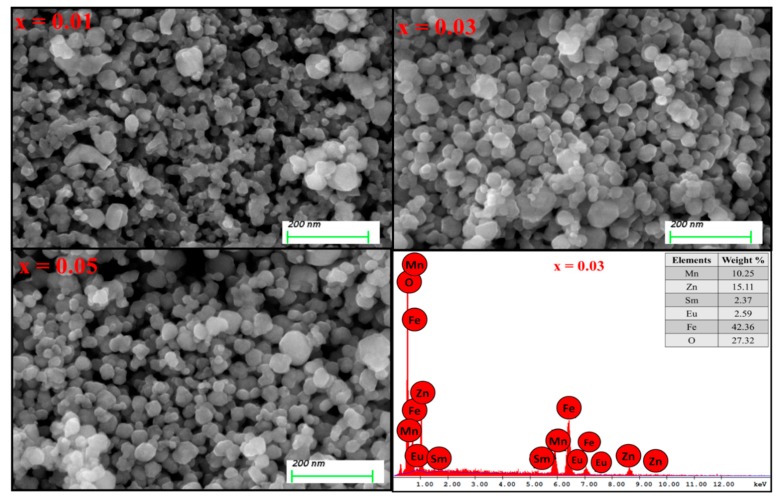
SEM of Mn_0.5_Zn_0.5_Eu_x_Sm_x_Fe_1.5−2x_O_4_ for x = 0.01, 0.03, and 0.05 and EDX x = 0.03 NPs.

**Figure 3:**
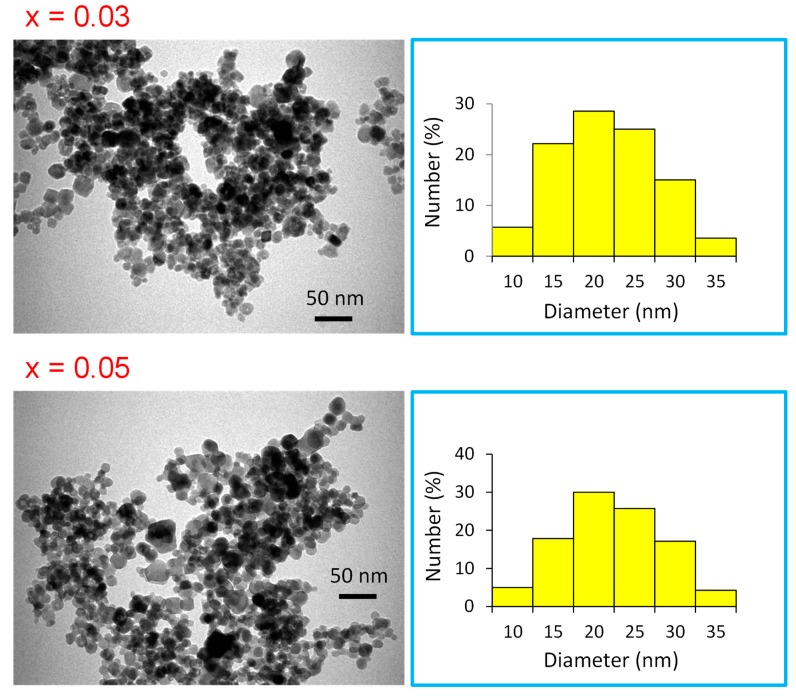
TEM of Mn_0.5_Zn_0.5_Eu_x_Sm_x_Fe_1.5−2x_O_4_ (x = 0.03 and 0.05) NPs.

XRD powder patterns of Mn_0.5_Zn_0.5_Eu_x_Sm_x_Fe_1.5−2x_O_4_ (0.01 ≤ x ≤ 0.05) NPs are offered in Figure 1. It is clear that all peaks belonged to a single phase of Mn-Zn spinel ferrite and no other peaks for the extra phase could be observed. This is evidence that the substituted ions were merged successfully into the spinel lattice. The lattice parameters and crystallite size were estimated by full proof refinement [26,27,28]. The lattice parameter “a” was 8.463, 8.434, 8.428, 8.413, and 8.408, respectively. It is obvious that the lattice parameters decreased when the ratio of Sm and Eu ions increased because of the substitutions of some Fe ions by larger ionic radii ions. The average of the crystallite sizes is in the range 7–12 nm. Figure 2 presents the SEM images of Mn_0.5_Zn_0.5_Eu_x_Sm_x_Fe_1.5−2x_O_4_ (x = 0.01, 0.03, and 0.05). All samples exhibited an agglomerated spherical particle. EDX was used to approve the stoichiometric composition spinel ferrite. The TEM of Mn_0.5_Zn_0.5_Eu_x_Sm_x_Fe_1.5−2x_O_4_ (x = 0.03 and 0.05) spinel ferrites displayed a spherical particle, as seen in Figure 3. It is well known that the complex 3d-metal oxides easily allow oxygen excess and/or deficit. Oxygen nonstoichiometry greatly affects the magnetic and magnetoelectric properties of complex oxides. Oxygen nonsctoichiometry changes the oxidation degree of 3d-metalls and magnetic parameters, such as the total magnetic moment and Curie point. The intensity of the exchange interactions decreases with the oxygen vacancy concentration increase. Exchange near the oxygen vacancies is negative according to Goodenough–Kanamori empirical rules [29,30].

### 3.2. Anti-Proliferative Activities

#### 3.2.1. Cell Proliferation Testing by MTT Assay

Antiproliferative activities of Mn_0.5_Zn_0.5_Sm_x_Eu_x_Fe_1.8−2x_O_4_ (0.01 ≤ x ≤ 0.05) NPs on cancerous cells were done by the MTT assay. After 48 h of treatment, the cytotoxic effects of Mn_0.5_Zn_0.5_Sm_x_Eu_x_Fe_1.8−2x_O_4_ (0.01 ≤ x ≤ 0.05) NPs were observed and we found that Mn_0.5_Zn_0.5_Sm_x_Eu_x_Fe_1.8−2x_O_4_ (0.01 ≤ x ≤ 0.05) NPs showed inhibitory action on HCT-116 cancerous cells. The inhibitory concentration (IC_50_) values of different compounds were calculated as depicted in Table 1. We also examined the effects of NPs on normal cells (HEK-293) to check whether they produced any cytotoxic effects. We found that NPs did not produce any significant cytotoxic effects on HEK-293 cells after 48 h of treatment.

#### 3.2.2. Nuclear Disintegration by Mn_0.5_Zn_0.5_Sm_x_Eu_x_Fe_1.8−2x_O_4_ (0.01 ≤ x ≤ 0.05) NPs Treatment

**Figure 4:**
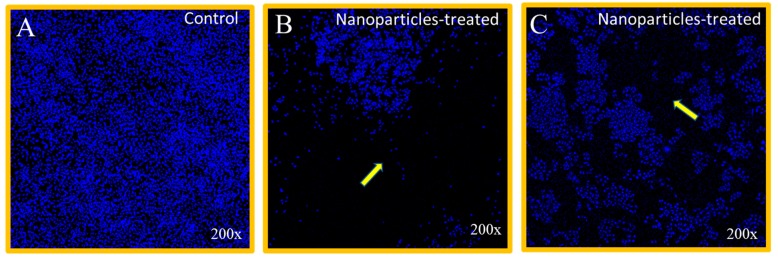
Cancer cells stained with **4′,6-diamidino-2-phenylindole** (DAPI). HCT-116 cells treated with Mn_0.5_Zn_0.5_Sm_x_Eu_x_Fe_1.8−2x_O_4_ (0.01 ≤ x ≤ 0.05) NPs for 48 h. (**A**) is control without treatment, (**B**) treated with Mn_0.5_Zn_0.5_Sm_x_Eu_x_Fe_1.8−2x_O_4_ NPs for x = 0.01 concentration (2.35 µg/mL), and (**C**) treated with Mn_0.5_Zn_0.5_Sm_x_Eu_x_Fe_1.8−2x_O_4_ NPs for x = 0.05 concentration (2.33 µg/mL). Arrows in B and C indicate the loss of nuclear staining. 200× magnifications.

The cancer cell nuclear morphology was evaluated by confocal scanning microscopy, which revealed that treatment of NPs showed strong inhibitory action on HCT-116 cells (Figure 4B,C) as compared to control group cells (Figure 4A).

There are several reports where magnetic nanoparticles have shown potential applications in drug delivery and other diagnostic assays [31,32,33,34]. These results suggest that NPs selectively targeted both colon and breast cancerous cells and do not cause any harm to normal and healthy cells. There are reports of involvement of nanoparticles in cancer cell death where nuclear fragmentation and nuclear disintegration were prominent features [35,36,37,38,39,40]. We recommend that Mn_0.5_Zn_0.5_Sm_x_Eu_x_Fe_1.8−2x_O_4_ (0.01 ≤ x ≤ 0.05) NPs possess a selective targeting capability to cancerous cells and could be a potential candidate for cancer treatments.

### 3.3. Antibacterial Study

#### 3.3.1. Antibacterial Activity (MIC/MBC)

**Figure 5:**
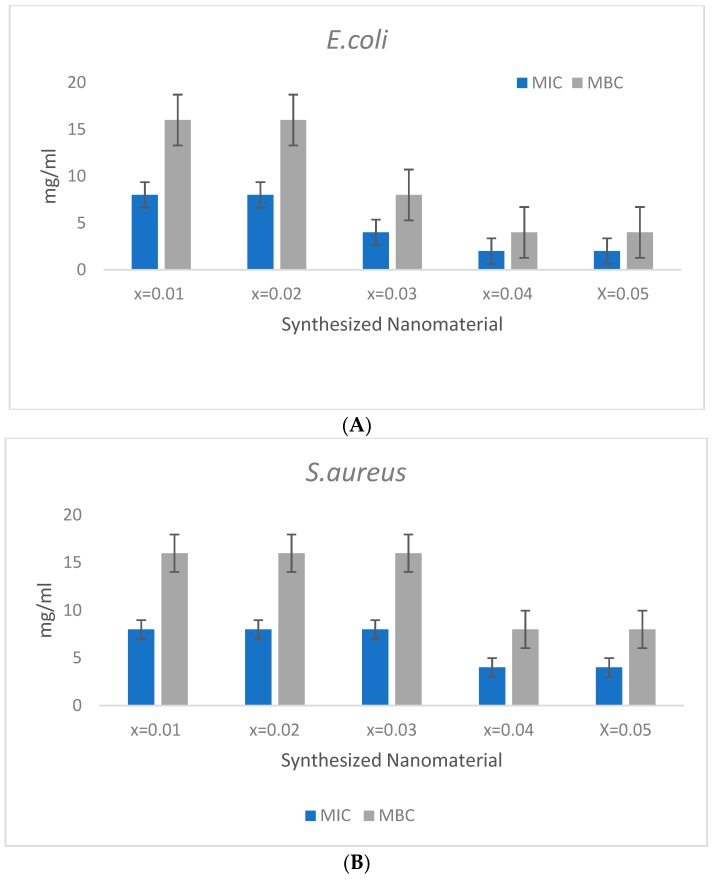
MIC/MBC of Mn_0.5_Zn_0.5_Sm_x_Eu_x_Fe_1.8−2x_O_4_ (0.01 ≤ x ≤ 0.05) against (**A**) *E. coli* (**B**) *S. aureus.*

The antibacterial activity of Mn_0.5_Zn_0.5_Sm_x_Eu_x_Fe_1.8−2x_O_4_ (0.01 ≤ x ≤ 0.05) NPs was evaluated by determining the MIC/MBC values. The nanoparticles were tested at the concentration ranging from of 16 to 0.5 mg/mL. For *E. coli*, the MIC/MBC values obtained were in the range of 8/16, 8/16, 4/8, 2/4, and 2/4 mg/mL for x = 0.01, 0.02, 0.03, 0.04, and 0.05, respectively (Figure 5A). The MIC/MBC values for *S. aureus,* were also found in the range of 8/16, 8/16, 8/16, 4/8, and 4/8 for x = 0.01, 0.02, 0.03, 0.04, and 0.05 mg/mL, respectively (Figure 5B). The activity of the broth culture was determined as the effectiveness of the content of element Fe (x = n) in the test material. The obtained results demonstrated that the effectiveness of the test material showed little improvement with the increasing ratio of the Fe content, i.e., the minimum MIC/ MBC was obtained by Fe x = 0.05 and 0.06. However, an enhanced activity was found against *E. coli* as compared to *S. aureus*; this slight difference could be attributed to the varying cell wall composition among these bacterial strains [41]. In some earlier studies, various metal-substituted NPs, like copper, zinc, nickel, and manganese, were recorded as possessing antibacterial activities [42,43], although the antibacterial activity of the current combination of Mn_0.5_Zn_0.5_Sm_x_Eu_x_Fe_1.8−2x_O_4_ (0.01 ≤ x ≤ 0.05) NPs is the first of its kind to the best of the author′s knowledge.

#### 3.3.2. Effects of Mn_0.5_Zn_0.5_Sm_x_Eu_x_Fe_1.8−2x_O_4_ (0.01 ≤ x ≤ 0.05) NPs on the Morphology of Bacteria

**Figure 6:**
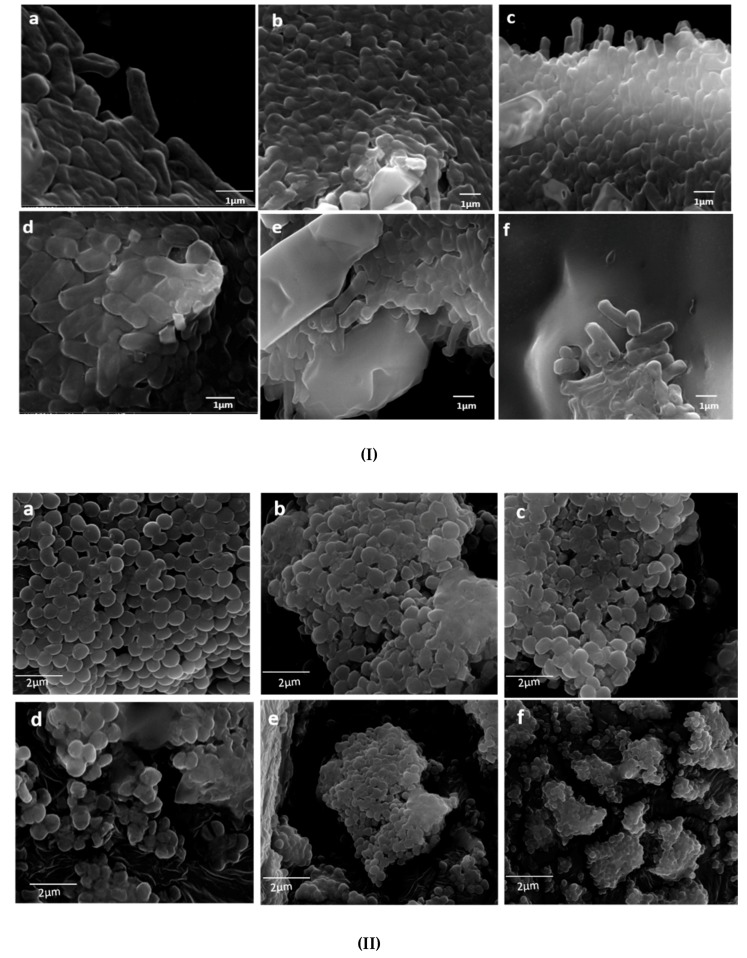
SEM micrographs of Mn_0.5_Zn_0.5_Sm_x_Eu_x_Fe_1.8−2x_O_4_ (0.01 ≤ x ≤ 0.05) NPs treated **(I)** *E. coli*, **(II)** *S. aureus.* (**a**) untreated cells (control), (**b**) x = 0.01, (**c**) 0.02, (**d**) 0.03, (**e**) 0.04, and (**f**) 0.05.

The morphological alteration in *E coli* and *S. aureus* cells caused by Mn_0.5_Zn_0.5_Sm_x_Eu_x_Fe_1.8−2x_O_4_ (0.01 ≤ x ≤ 0.05) NPs was further studied by SEM. The untreated (control) cells of both organisms were normal in shape, and intact with a regular and smooth cell surface (Figure 6I). However, cells treated with Mn_0.5_Zn_0.5_Sm_x_Eu_x_Fe_1.8−2x_O_4_ (x ≤ 0.05) NPs were not found intact, i.e., the cells started appearing abnormal in shape with irregularities at the cell surfaces (Figure 6(Ib–If)). During the examination, untreated cells had no obvious visible damage, but the treated cells were observed as moderately damaged to severely damaged, with the increasing ratio of Fe_(x=n)_, i.e., the maximum damage was caused by x = 0.04 and x = 0.05. Furthermore, it was observed that the effect of Mn_0.5_Zn_0.5_Sm_x_Eu_x_Fe_1.8−2x_O_4_ (x ≤ 0.05) NPs on both gram positive and gram negative were somehow similar, although a slightly enhanced activity was observed against *E. coli* (Figure 6I,II). This difference in activity can be attributed to the varying cell wall composition between the two organisms, which in turn may play an important role in the attachment of Mn_0.5_Zn_0.5_Sm_x_Eu_x_Fe_1.8−2x_O_4_ (x ≤ 0.05) NPs to the bacterial cell surface, and is therefore essential in obtaining enhanced antibacterial activity [44].

## 4. Conclusions

In the present study, we synthesized five different derivatives of Mn_0.5_Zn_0.5_Sm_x_Eu_x_Fe_1.8−2x_O_4_ (0.01 ≤ x ≤ 0.05) NPs hydrothermally. The structure and surface morphology of Mn_0.5_Zn_0.5_SmEuFe_1.8−2x_O_4_ spinel hydrothermal NPs were characterized by the XRD, SEM, TEM, and EDX methods, respectively. We examined the impact of Mn_0.5_Zn_0.5_Sm_x_Eu_x_Fe_1.8−2x_O_4_ (0.01 ≤ x ≤ 0.05) NPs on both normal (HEK-293) and cancerous (HCT-116) cells. We found that after 48 h of treatment, Mn_0.5_Zn_0.5_Sm_x_Eu_x_Fe_1.8−2x_O_4_ (0.01 ≤ x ≤ 0.05) NPs showed a significant decline in the cancer cell population. The IC_50_ value of Mn_0.5_Zn_0.5_Sm_x_Eu_x_Fe_1.8−2x_O_4_ (0.01 ≤ x ≤ 0.05) NPs ranged between 0.75 to 2.25 µg/mL. When tested on normal and healthy cells (HEK-293), we found that the treatment of Mn_0.5_Zn_0.5_Sm_x_Eu_x_Fe_1.8−2x_O_4_ (0.01 ≤ x ≤ 0.05) NPs did not produce any effects on normal cells, which suggests that Mn_0.5_Zn_0.5_Sm_x_Eu_x_Fe_1.8−2x_O_4_ (0.01 ≤ x ≤ 0.05) NPs selectively targeted cancerous cells. The anti-bacterial properties of Mn_0.5_Zn_0.5_Sm_x_Eu_x_Fe_1.8−2x_O_4_ (0.01 ≤ x ≤ 0.05) NPs were also evaluated by MIC and MBC assays. We conclude that Mn_0.5_Zn_0.5_Sm_x_Eu_x_Fe_1.8−2x_O_4_ (0.01 ≤ x ≤ 0.05) NPs produced via the hydrothermal route possess potential anti-cancer and anti-bacterial abilities.

## Figures and Tables

**Table 1:** Effect of Mn_0.5_Zn_0.5_SmEuFe_1.8−2x_O_4_ (0.01 ≤ x ≤ 0.05) NPs on cancerous cells **human colon carcinoma cells** (HCT-116) and normal cells **human embryonic kidney cells** (HEK-293).

x	IC_50_ (HCT-116) (µg/mL)	IC_50_ (HEK-293)
0.01	0.75 µg/mL	No inhibition
0.02	0.85 µg/mL	No inhibition
0.03	2.25 µg/mL	No inhibition
0.04	0.88 µg/mL	No inhibition
0.05	0.79 µg/mL	No inhibition

Note: IC_50_ Value [µg/mL] = Inhibitory concentration (IC).
